# 22. International Multicenter Study Comparing Cancer to Non-Cancer Patients with COVID-19: Impact of Risk Factors and Treatment Modalities on Outcome

**DOI:** 10.1093/ofid/ofab466.022

**Published:** 2021-12-04

**Authors:** Ray Y Hachem, Anne-Marie Chaftari, Nigo Masayuki, Nelson Hamerschlak, Hiba Dagher, Ying Jiang, Bilal Siddiqui, Arnaud Bayle, Robert Somer, Ana Fernandez Cruz, Edward Gorak, Arvinder Bhinder, Nobuyoshi Mori, Tarcila Datoguia, Samuel Shelanski, Tomislav Dragvich, Yee Elise Vong Kiat, Suha Fakhreddine, Pierre Abi Hanna, Roy F Chemaly, Victor E Mulanovich, Javier Adachi, Jovan Borjan, Fareed Khawaja, Bruno Granwehr, Teny John, Eduardo Yepez Guevara, Harrys A Torres, Monica Slavin, Benjamin Teh, Vivek Subbiah, Dimitrios P Kontoyiannis, Alexandre Malek, Issam I Raad

**Affiliations:** 1 MD Anderson Cancer Center, Houston, TX; 2 The University of Texas MD Anderson Cancer Center, Houston, Texas; 3 University of Texas in Houston, Houston, TX; 4 Hospital Israelita Albert Einstein, São Paulo, Brasil, Sao Paulo, Not Applicable, British Indian Ocean Territory; 5 UT MD Anderson Cancer Center, Houston, Texas; 6 Communty Health Network, Indiannapolis, Indiana; 7 Gustave Roussy Cancer Hospital, Villejuif, Lorraine, France; 8 Cooper University Healthcare, Camden, New Jersey; 9 Unidad de Enfermedades Infecciosas, Madrid, Madrid, Spain; 10 Baptist MD Anderson Cancer Center, Jacksonville, Florida; 11 OhioHealth Physician Group, Marion, Ohio; 12 St. Luke's International Hospital, Tokyo, Tokyo, Japan; 13 Hospital Israelita Albert Einstein, Sao Paulo, Sao Paulo, Brazil; 14 Banner MD Anderson at Mckee Medical Center, Loveland, Colorado; 15 Banner MD Anderson Cancer, Gilbert, Arizona; 16 Tan Tock Seng Hospital, Novena, Not Applicable, Singapore; 17 Rafik Hariri University Hospital, Beirut, Beqaa, Lebanon; 18 University of Texas MD Anderson Cancer Center, Houston, TX; 19 The University of Texas MD Anderson Cancer Center, Houston, Texas, Houston, TX; 20 National Centre for Infections in Cancer, Peter MacCallum Cancer Centre, Melbourne, Victoria, Australia; 21 Peter MacCallum Cancer Centre, Melbourne, Victoria, Australia

## Abstract

**Background:**

Given the limited collaborative international studies that evaluated COVID-19 in patients with cancer in comparison to patients without cancer, we aimed to determine the independent risk factors associated with increased 30-day mortality and the impact of novel treatment modalities in a large group of cancer and non-cancer patients with COVID-19 from multiple countries.

**Methods:**

We retrospectively collected de-identified data on cancer and non-cancer patients diagnosed with COVID-19 between January and November 2020, at 16 centers in Asia, Australia, Europe, North America, and South America. A logistic regression model was used to identify independent predictors of all-cause mortality within 30 days after COVID-19 diagnosis.

**Results:**

Of the total 4015 COVID-19 confirmed patients entered, we analyzed 3966 patients, 1115 cancer and 2851 non-cancer patients. Cancer patients were older than non-cancer patients (median age, 61 vs 50 years; p< 0.0001); more likely to be pancytopenic , had pulmonary disorders, hypertension, diabetes mellitus. In addition, they were more likely to present with higher inflammatory biomarkers (D-dimer, ferritin and procalcitonin), but were less likely to present with clinical symptoms. By multivariable logistic regression analysis, cancer was an independent risk factor for 30-day mortality (OR 1.46; 95% CI 1.03 to 2.07; p=0.035). Older age (≥65 years) was the strongest predictor of 30-day mortality in all patients (OR 4.55; 95% CI 3.34 to 6.20; p< 0.0001). Remdesivir was the only therapeutic agent independently associated with decreased 30-day mortality (OR 0.58; CI 0.39-0.88; p=0.009). Among patients on low-flow oxygen at admission, patients who received remdesivir had a lower 30-day mortality rate than those who were on high flow oxygen (5.9% vs 17.6%; p=0.03). Patients transfused with convalescent plasma within 1 day of diagnosis had a lower 30-day mortality rate than those transfused later (1% vs 7%, *p*=0.04).

**Conclusion:**

Cancer is an independent risk factor for increased 30-day all-cause mortality from COVID-19. Remdesivir, particularly in patients receiving low-flow oxygen, can reduce 30-day all-cause mortality, as well as convalescent plasma given early after COVID-19 diagnosis.

**Disclosures:**

**Roy F. Chemaly, MD, MPH, FACP, FIDSA**, **AiCuris** (Grant/Research Support)**Ansun Biopharma** (Consultant, Grant/Research Support)**Chimerix** (Consultant, Grant/Research Support)**Clinigen** (Consultant)**Genentech** (Consultant, Grant/Research Support)**Janssen** (Consultant, Grant/Research Support)**Karius** (Grant/Research Support)**Merck** (Consultant, Grant/Research Support)**Molecular Partners** (Consultant, Advisor or Review Panel member)**Novartis** (Grant/Research Support)**Oxford Immunotec** (Consultant, Grant/Research Support)**Partner Therapeutics** (Consultant)**Pulmotec** (Consultant, Grant/Research Support)**Shire/Takeda** (Consultant, Grant/Research Support)**Viracor** (Grant/Research Support)**Xenex** (Grant/Research Support) **Fareed Khawaja, MBBS**, **Eurofins Viracor** (Research Grant or Support) **Monica Slavin, MBBS,MD**, **F2G** (Advisor or Review Panel member)**Merck** (Advisor or Review Panel member)**Pfizer** (Advisor or Review Panel member) **Dimitrios P. Kontoyiannis, MD**, **Astellas** (Consultant)**Cidara Therapeutics** (Advisor or Review Panel member)**Gilead Sciences** (Consultant, Grant/Research Support, Other Financial or Material Support, Honoraria)

